# Reptiles from Lençóis Maranhenses National Park, Maranhão, northeastern Brazil

**DOI:** 10.3897/zookeys.246.2593

**Published:** 2012-11-29

**Authors:** Jivanildo Pinheiro Miranda, João Carlos Lopes Costa, Carlos Frederico D. Rocha

**Affiliations:** 1 Pós-Graduação em Ecologia, Universidade Estadual de Campinas, Instituto de Biologia, Caixa Postal 6109, CEP 13084-970, Campinas, SP, Brazil; Current Address: Universidade Federal do Maranhão, Centro de Ciências Agrárias e Ambientais, MA-230, Km 4, S/N, CEP 65500-000, Chapadinha, MA, Brazil; 2 Pós-graduação em Zoologia, Laboratório de Herpetologia, Museu Paraense Emilio Goeldi, Avenida Magalhães Barata, 376, Terra Firme, CEP 66040-170, Belém, PA, Brazil; 3 Universidade do Estado do Rio de Janeiro, Departamento de Ecologia, Rua São Francisco Xavier, 524, CEP 20550-011, Rio de Janeiro, RJ, Brazil

**Keywords:** Richness, ecotone, lizards, snakes, turtles, worm lizards, dunes, restingas

## Abstract

We are presenting a list of the reptile species from Lençóis Maranhenses National Park (LMNP), Maranhão, Brazil, obtained during 235 days of field work. The study area is located in the contact zone between three major Neotropical ecosystems: Amazonia, Caatinga, and Cerrado. The PNLM encompasses the largest dune fields in Brazil, wide shrubby areas (restingas), lakes, mangroves, and many freshwater lagoons. We have recorded 42 species of reptiles in the area: 24 snakes, 12 lizards, two worm lizards, three turtles, and one alligator. About 81 % of the recorded species occurred only in restinga areas. Our data highlights the uniqueness of the PNLM in the context of the biomes that surround it and shows the importance of efforts to improve the conservation of reptiles living in the restinga, which currently comprise only about 20 % of the total area protected by the park, but which are the mesohabitat containing most of the reptile species in the Lençóis Maranhenses complex of habitats.

## Abstract (es)

No presente estudo apresentamos uma lista das espécies de répteis presentes no Parque Nacional dos Lençóis Maranhenses (LMNP), Maranhão, Brazil. A área do Parque Nacional dos Lençóis Maranhenses está localizada em um complexo ecótono na zona de contato entre a Amazônia, a Caatinga e o Cerrado. Além desta localização singular, a área inclui o maior campo de dunas do Brasil, extensas restingas, lagos, manguezais e uma grande quantidade de lagoas de água doce, formadas nos vales do campo de dunas. Registramos 42 espécies de répteis: 24 serpentes, 12 lagartos, duas cobras-cegas, três quelônios e um jacaré. Destas, cerca de 81 % foram encontrados apenas nos ambientes de restinga. Os resultados apresentados aqui enfatizam a singularidade do PNLM no contexto dos biomas que o cercam e ressaltam a importância de ações para incrementar a conservação das áreas de restinga, as quais, atualmente, constituem apenas 20 % da área total protegida pelo parque, mas constituem o mesohábitat onde ocorre a maioria das espécies de répteis que vivem no complexo de ambientes que compõe os Lençóis Maranhenses.

## Introduction

Brazil is a megadiverse country, including six biomes (Ab’Sáber 2003). One of its most distinguishable regions is the complex of ecotones adjacent to the Amazon forest of northern Brazil, the Caatinga in the northeastern portion of the country, and the Cerrado of central Brazil (Ab’Sáber 2003; Olson et al. 2005, Rodrigues 2005). In the very heart of this transitional region, one of the most remarkable environments is a region known as Lençóis Maranhenses in the state of Maranhão, located in the northeast of Brazil. The Lençóis Maranhenses comprises an unexpected and admirable landscape composed of the largest dunefields in Brazil (about 120,000 ha of continuous sand dunes), which is scattered by thousands of freshwater lagoons formed annually by rainfalls. In 1981, the area was converted into a park called Lençóis Maranhenses National Park due to its amazing scenery. Despite its relevance as a unique ecosystem in that transitional zone, animal and plant components are still poorly known. The few available studies just focused on individual species, usually only reporting its occurrence (*e. g*. Rêgo and Albuquerque 2006). There is no published inventory of the flora and fauna of Lençóis Maranhenses National Park. Regarding the fauna of reptiles in the park, the only study we are aware of is a description of a new turtle species by (Vanzolini 1995). The lack of a species list restricts the knowledge of the reptile species composition in the area. This hampers specific conservation efforts to protect local species diversity. Herein, as a result of almost two years of study conducted in the area, we are providing a report of reptile species composition for the Lençóis Maranhenses National Park, a detailed description of the main mesohabitats in the area, and some suggestions to improve the conservation of reptiles in the park.

## Materials and methods

### Study site

The Lençóis Maranhenses National Park (LMNP) is located in the Northeastern coast of Brazil (central coordinates: 02°31'02"S, 43°01'54"W, SAD69). The area of the park (about 155,000 hectares) is composed of sand dunes, freshwater lagoons, restingas (local name for herbaceous and shrubby vegetation), lakes, mangroves, and 70 km of beach. The dunefields arose from the varieties of sediments due to retrogradations from sedimentary deposits (Barreiras formation of Tertiary age), the correspondent widening of the continental shelf, successive marine transgressions since Pleistocene, and inputs of fluvial sediments from the main rivers in that region (Castro and Piorski 2002).

The climate in LMNP is warm (mean annual temperature: 28.5°C) with relatively little temperature variation throughout the year (about 1.1 °C, in average) and an annual rainfall between 1,600 and 2,400 millimeters (Nimer 1989, Castro and Piorski 2002). Most of the rain (about 70 %) occurs from January to May, when the level of underground water supply rises, and seasonally surfaces in the spaces between successive dunes, as lagoons.

**Figure 1.**
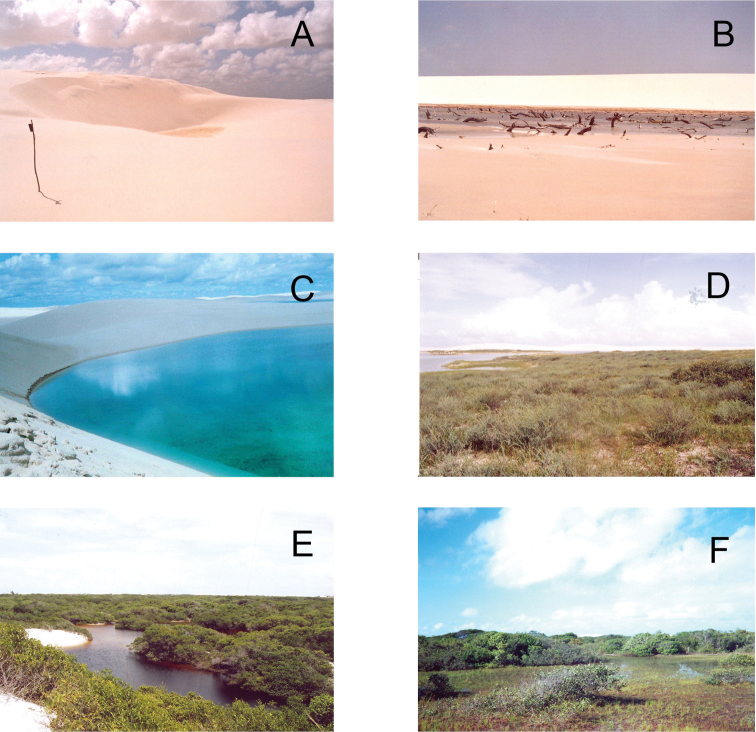
Mesohabitats found at Lençóis Maranhenses National Park, Maranhão State, Northeastern Brazil: **A** Sand dunes “Morrarias” **B** Remains of restinga vegetation buried by sand transportation **C** Freshwater lagoon **D** “Vargem” **E** Restinga mosaics in the boundary of sand dunes, including a lagoon **F** Restinga mosaics far away from the sand dunes. Photos by J. P. Miranda.

**Figure 2.**
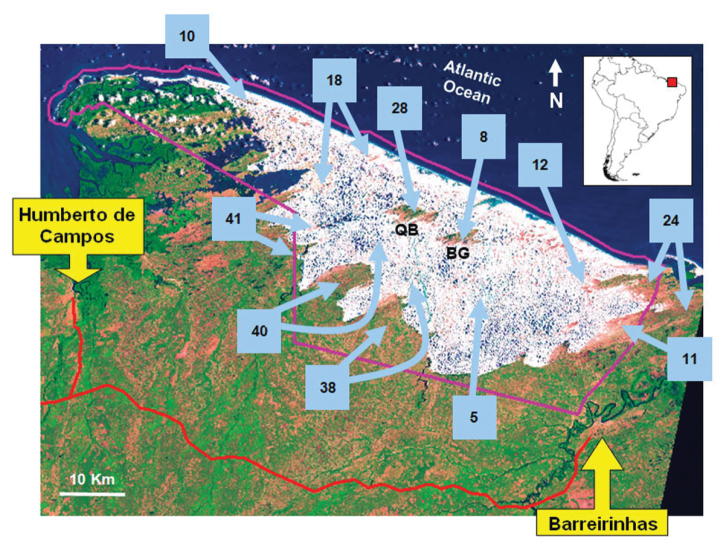
Satellite image Landsat showing sample sites (light blue arrows) in the region of the Lençóis Maranhenses National Park, Maranhão State, Northeastern Brazil. The amount of sampling days is specified inside light blue squares. Two arrows linked to the same squares and pointing to different places indicate that the sampling effort in the square is divided between these sites. White areas with blue spots are sand dunes and freshwater lagoons, respectively. The greenish areas flecked by the orange color, are restingas habitats (green represents shrubby areas and orange most opening areas). QB and BG indicate the position of the isolated restingas called Queimada dos Britos and Baixa Grande, respectively. The violet line indicates the territory of LMNP. The red line is the road that accesses the park (MA-402). The location of the provinces Barreirinhas and Humberto de Campos are provided in the yellow rectangles. The position of the park in South America is provided upper right. Satellite image modified after Castro and Piorski (2002).

We have distinguished the following seven mesohabitats in the LMNP: **1) Sand dunes “Morrarias”** (vernacular expression): are sand dunes with no stabilizing vegetation, which is the most frequent and dynamic mesohabitat in the park (Figure 1A). The constant movement of sand dunes influences all other mesohabitats. The transportation of sand by wind constantly buries the vegetation in the area bordering the dunes (Figure 1B). Additionally, the migration of contiguous dunes spill water from one lagoon to the next closest one, resulting in a high interchange of water among lagoons. During the rainy season, however, the migration of dunes is slower because of the moisture that avoids sand transference (Parteli et al. 2006); **2) Freshwater lagoons:** every year, thousands of freshwater lagoons appear in the dune fields in LMNP (Figure 1C). In the rainy season, lagoons can cover up to 41 % of the total area of the park (Levin et al. 2006), which represents about 64,000 ha. Most freshwater lagoons are shallow (less than 1m deep), and therefore are temporary. However, as reported to us by the native people, some rare lagoons can be as old as 16-20 years. Aquatic macrophytes like *Utricularia* sp. (Lentibulariaceae) and many species of algae can be found in freshwater lagoons; **3) Vargem** (vernacular expression)**:** are plain areas located in the depressions between successive dunes where there are herbaceous plants called “vassoura” (Figure 1D). The “vassoura” vegetation is composed mainly of plants of the genera *Cassia* (Fabaceae) and *Borreria* (Rubiaceae), which normally grow in areas where freshwater lagoons have dried up; **4) Restingas:** are mosaics of open areas, freshwater lagoons, with herbaceous and shrubby vegetation (Figure 1E). The shrubby vegetation is composed mainly of grasses (Poaceae) and of “mirim” and “guajirú”, which are plants belonging to the genera *Humiria* (Humiriaceae) and *Chrysobalanus* (Chrysobalanaceae) (Figure 1F). Restinga areas can be found within the park and in neighboring areas. These neighboring restinga areas are considered buffer zones around the park; **5) Innermost Isolated Restingas:** In the middle of the dune field there are two “oases” (isolated restingas), one is called Queimada do Britos and the other is Baixa Grande. Queimada do Britos is the largest one, which is about 1,100 hectares. Baixa Grande has an area of 850 hectares and becomes largely flooded during the rainy season (Figure 2); **6) Rivers:** There are many rivers and creeks in the region of the park. At least two of them connect to the fresh water lagoons during the rainy season: Rio Grande (in the region of Lagoa da Betânia) and Rio Negro (in the region of Lagoa da Esperança), which in the years of high precipitation, cross the dunefield and reach the Atlantic Ocean; **7) Beaches**: The coastal area of the park consists of 70 km of beaches. At these beaches it is common to find ropes, bottles and other human materials, which are constantly carried to the beach by the sea.

### Data collection

The species survey of reptiles at LMNP was conducted from September, 2004 to April, 2006 (IBAMA permit number 02001.004089/03-50). In that period, we made 47 trips, totaling 235 days of field sampling. At each site (see Figure 2), we sampled about three hectares. We used the time-constrained sample method, which is performed by walking slowly and searching for specimens in all visually accessible microhabitats (Heyer et al. 1994). The sampling effort was calculated during several days of sampling. Each day of sampling equaled the efforts of two collectors. These two collectors searched for specimens from 09:00 to 15:00 h (6 hours during the day) and from 19:00 to 23:00 h (4 hours during the night), totaling 1,410 hours of diurnal sampling and 940 hours of nocturnal sampling. Occasionally, additional specimens were obtained by random encounters, or were provided by the local people. Voucher specimens were deposited in the Coleção Herpetológica “Claude d’Abbeville” (CHMA) at Universidade Federal do Maranhão, Chapadinha, Maranhão, Brazil. The nomenclature of species follows the proposed taxonomy of Zaher et al. (2009) and Grazziotin et al. (2012) for families Colubridae and Dipsadidae, Hedges and Conn (2012) for family Mabuyidae, and Harvey et al. (2012) for Teiidae. Other families follow the nomenclature of the Brazilian List of Reptiles Species (SBH 2012).

### Data analysis

We constructed species-accumulation curves that were generated using the nonparametric binomial mixture model of Mao et al. (2005). Additionally, to estimate the overall predicted species richness (extrapolation) for each reptile grouping (lizards or snakes), we used the first order Jackknife estimator (Heltshe and Forrester 1983). All analyses were made with EstimateS 8.0 (Colwell 2007). The results of Jackknife estimator appear within ± 1 standard deviation.

## Results

We recorded 42 reptile species in the LMNP: 12 species of lizards, belonging to 11 genera and eight families (Gekkonidae, Sphaerodactylidae, Mabuyidae, Gymnophthalmidae, Iguanidae, Polychrotidae, Teiidae and Tropiduridae); two species of worm lizards belonging to the genus *Amphisbaena*, in the family Amphisbaenidae; 24 species of snakes, belonging to 20 genera and four families (Boidae, Colubridae, Dipsadidae and Elapidae); three species of turtles, belonging to three genera and three families (Cheloniidae, Dermochelyidae, and Emydidae); and one species of alligator (Alligatoridae) (Table 1).

**Table 1.** Reptile species recorded at Lençóis Maranhenses National Park, Maranhão State, Northeastern Brazil, respective environments of occurrence in the park, and figure numbers (when available). In the column mesohabitat, the numbers correspond to the following mesohabitats: 1) Sand dunes “Morrarias”; 2) Freshwater lagoons; 3) Vargem; 4) Restingas; 5) Innermost isolated restingas; 6) Rivers; 7) Beaches.<br/>

**Reptilia**	**Mesohabitat**	**Figure**
OrderSAURIA		
**Family Sphaerodactylidae**		
*Gonatodes humeralis* (Guichenot, 1855)	4	5A
**Family Gekkonidae**		
*Hemidactylus mabouia* (Moreau de Jonnès, 1818)	4, 5	5B
**Family Gymnophthalmidae**		
*Colobosaura modesta* (Reinhardt and Lütken, 1862)	4	
**Family Iguanidae**		
*Iguana iguana* (Linnaeus, 1758)	4, 5	5C
**Family Mabuyidae**		
*Varzea bistriata* (Spix, 1825)	4, 5	5D
*Brasiliscincus heathi* (Schmidt and Inger, 1951)		5E
**Family Polychrotidae**		
*Polychrus acutirostris* Spix, 1825	4	5F
**Family Teiidae**		
*Ameiva ameiva* (Linnaeus, 1758)	4, 5	5G
*Ameivula ocellifera* (Spix, 1825)	1, 4, 5	5H
*Kentropyx calcarata* Spix, 1825	4	6A
*Tupinambis teguixin* (Linnaeus, 1758)	1, 4, 5	
**Family Tropiduridae**		
*Tropidurus hispidus* (Spix, 1825)	1, 4, 5	6B
OrderAMPHISBAENIA		
**Family Amphisbaenidae**		
*Amphisbaena ibijara* Rodrigues, Andrade & Lima, 2003	4	
*Amphisbaena vermicularis* Wagler, 1824	4	6C
OrderSERPENTES		
**Family Boidae**		
*Boa constrictor* Linnaeus, 1758	4	6D
*Eunectes murinus* (Linnaeus, 1758)	4, 5	
**Family Colubridae**		
*Chironius flavolineatus* (Jan, 1863)	4	
*Drymarchon corais* (Boie, 1827)	4, 5	6E
*Leptophis ahaetulla* (Linnaeus, 1758)	4, 5	6G
*Mastigodryas bifossatus* (Raddi, 1820)	4, 5, 6	
*Oxybelis aeneus* (Wagler, 1824)	4	6H
*Oxybelis fulgidus* (Daudin, 1803)	4	7A
*Spilotes pullatus* (Linnaeus, 1758)	4	
*Tantilla melanocephala* (Linnaeus, 1758)	4	
**Family Dipsadidae**		
*Helicops angulatus* (Linnaeus, 1758)	4, 5, 6	6F
*Hydrodynastes gigas* (Duméril, Bribon and Duméril, 1854)	4, 6	
*Erythrolamprus poecilogyrus* (Wied-Neuwied, 1825)	1, 4, 5	7B
*Erythrolamprus taeniogaster* (Jan,1866)	4	
*Leptodeira annulata* (Linnaeus, 1758)	4	
*Lygophis meridionalis* (Schenkel, 1902)	4	
*Oxyrhopus trigeminus* Duméril, Bibron and Duméril, 1854	4, 5	7C
*Philodryas nattereri* Steindachner, 1870	4	7D
*Philodryas olfersii* (Lichtenstein, 1823)	4	
*Psomophis joberti* (Sauvage, 1884)	4	7E
*Taeniophallus occipitalis* (Jan, 1863)	4	
*Thamnodynastes hypoconia* (Cope, 1860)	4	7F
*Xenodon merremii* (Wagler, 1854)	4	7G
**Family Elapidae**		
*Micrurus ibiboboca* (Merrem, 1820)	4, 5	
OrderTESTUDINES		
**Family Cheloniidae**		
*Chelonia mydas* (Linnaeus, 1758)	7	
**Family Dermochelyidae**		
*Dermochelys coriacea* (Vandelli, 1761)	7	
**Family Emydidae**		
*Trachemys adiutrix* Vanzolini, 1995	1, 2, 3	7H
OrderCROCODYLIA		
**Family Alligatoridae**		
*Caiman crocodilus* (Linnaeus, 1758)	6	

**Figure 3.**
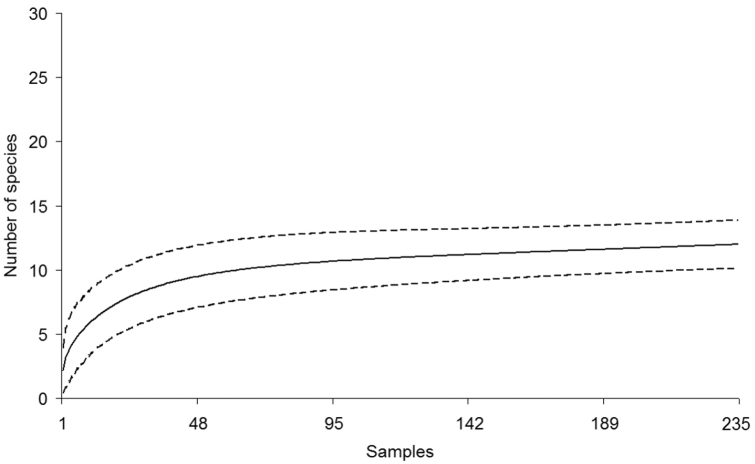
Accumulation curve for lizards recorded at the region of the Lençóis Maranhenses National Park (LMNP), Maranhão State, Northeastern Brazil (solid line). Dashed lines are confidence intervals at 95 %. The total number of sampling days is 235. A sample is equal to the search effort of two people looking for reptile species from 09:00 to 15:00 h and from 19:00 to 23:00 h.

**Figure 4.**
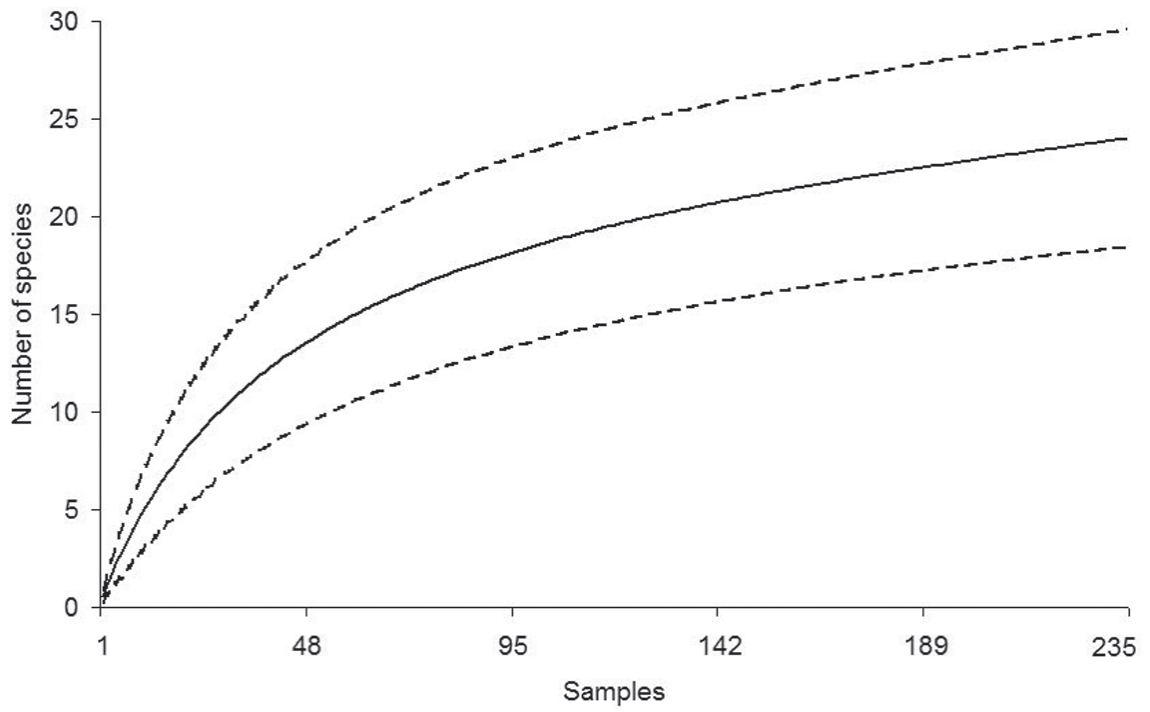
Accumulation curve for snakes recorded at the region of the Lençóis Maranhenses National Park (LMNP), Maranhão State, Northeastern Brazil (solid line). Dashed lines are confidence intervals at 95 %. The total number of sampling days is 235. A sample is equal to the search effort of two people looking for reptile species from 19:00 to 23:00 h and from 09:00 to 15:00 h.

The species-accumulation curves for snakes and lizards have different slopes and confidence intervals according to the reptile group studied. Nevertheless, both curves predicted more species than currently recorded. Richness for each reptile group was quite close to the predicted values, especially for the lizards (Figures 3 and 4). The first order Jackknife estimator predicted that 13 to 15 species of lizards [N(J1) = 13,99 ± 1,40], and 28 to 34 species of snakes [N(J1) = 30,97 ± 2,95] might be recorded in LMNP. Overall, most reptile species that we recorded at LMNP were found in the restingas (Table 1).

## Discussion

In the management plans of the LMNP, there is no list of Herpetofauna’s species (Castro and Piorski 2002). However, there is mention about the occurrence of the Brazilian slider turtle, *Trachemys adiutrix*, in the park. Thus, our study added 41 species to the number of reptiles known in the LMNP. Despite the uniqueness of the environment, and conditions in the LMNP, the reptile taxocenosis recorded there includes species normally found in the biomes that surround it. For instance, *Gonatodes humeralis* and *Varzea bistriata* which are associated with the Amazon, and *Philodryas nattereri*, *Polychrus acutirostris* and *Brasiliscincus heathi* which are typical Cerrado and Caatinga inhabitants. One remarkable exception is *Trachemys adiutrix*, which is endemic to a small area at the coast of Maranhão and Piauí (see Ávila-Pires 1995, Rodrigues and Prudente 2011, Mesquita et al. 2006, Vanzolini 1995 and references therein). Therefore, LMNP has a significant importance for conservation of reptiles in Brazil, as it includes in one relatively small area (155,000 ha), a single taxocenosis of reptiles that combine species from various Brazilian biomes, all in a very unique landscape.

In LMNP there are only three species of lizards: (*Tropidurus hispidus*, *Ameivula ocellifera*, and *Tupinambis teguixin*), one snake (*Erythrolamprus poecilogyrus*), and *Trachemys adiutrix* which were recorded in sand dune areas. Additionally, two sea turtles were recorded at the coastal area of the park (*Chelonia mydas* and *Dermochelys coriacea*). Thus, about 81 % (34 species) of reptile species recorded at LMNP were only found in restingas. In addition, the management plans of the LMNP emphasize the innermost isolated restingas for their actions in conservation, as opposed to restinga areas located at the southern LMNP (Castro and Piorski 2002). The importance of actions to protect the innermost isolated restingas is justified due to the high diversity of plant species found in those isolated areas (Castro and Piorski 2002). However, the reptile taxocenosis from the park did not follow that pattern of richness. The species recorded at the isolated restingas were only a subset of the reptile species registered at the restingas which are adjacent to sand dunes in the southern LMNP (see Table 1 and Figure 2).

During our field work, we observed that the restingas in the south area of the park (both inside the park and in the buffer zones) have been strongly disturbed by the clandestine openings of paths created to transport tourists to the dunes and lagoons in the park, using off-road vehicles. This problem is more severe during the rainy season (from January from June) when paths become muddy quickly, and new paths are continuously opening. Restinga are extremely sensitive to clearing because the poor soils hinder habitat recomposition (Hay et al. 1981). In fact, the restinga areas in the south of the park (outside LMNP) are included in an environmental protection area called APA Upaon Açu-Miritiba-Alto Preguiças, which is a type of protected area in the Brazilian system of conservation units which ensures sustainable use. However, the surveillance of this use is still very limited.

On the beach and sand dune areas, we recorded three species of turtles which are included in the IUCN Red List of Threatened Species (IUCN 2012): *Dermochelys coriacea* (critically endangered), *Chelonia mydas* (endangered), and *Trachemys adiutrix* (endangered). During our field work, we observed the *Trachemys adiutrix* (locally called “Pininga”) being used as a food source; eaten by the extremely poor human population which live in the park area and surroundings. These turtles cannot be seen very easily in LMNP during most of the year. However, as the lagoons begin to diminish (both in number and surface) in the dry season, these turtles concentrate in the few remaining lagoons, making it easy to capture a large number of individuals at the same time. Some people capture the turtles and keep them alive, in order to eat them during the dry season. This is the time when fish and other food items are scarce for the local human population.

In the sand dunes at LMNP, which is an extremely open area, the ground temperature can easily exceed 70° C during the warmest period of the day (JPM Pers. Obs.). This particular characteristic of sand dune areas reinforces the importance of shelters (dead branches and patches of vegetation) and burrows for the species that live there. For lizards, shelters and burrows are important for thermoregulation, as this is one of the few options to decrease exposure to the sun (Rocha et al. 2009). Furthermore, in sand dune areas at LMNP, there are some species of predatory birds like “carcará” (*Polyborus plancus*, Falconidae) and “caburé” (*Athene cunicularia*, Strigidae) (JPM Pers. Obs), known to prey on reptiles (Andrade et al. 2010, Vargas et al. 2007). Thus, shelters and burrows may also be important for the protection against predators. Whiptail lizards (*Ameivula ocellifera*) have a great ability to dig (see Eifler and Eifler 1998), which might have been important for their successful establishment in sand dunes. The other lizard species found in sand dunes, *Tropidurus hispidus*, is not able to dig as well, but is known as a species with great flexibility in habitat use (Ávila-Pires 1995). In sand dunes at LMNP, *Trachemys hispidus* is often sheltered in dead branches of the shrubs buried by sand. During our fieldwork, we often observed the clandestine traffic of off-road vehicles in the dunes, which can be harmful to the reptiles that live in that mesohabitat due to the fact that heavy vehicles destroy a large number of burrows and shelters used by those species. This would be similar to the adverse effects of off-road vehicles on lizard populations observed by Busack and Bury (1974) in the Mojave desert, USA.

The only exotic invasive reptile species at LMNP was the gecko *Hemidactylus mabouia*, which was found on different occasions in natural habitats and microhabitats within the study area. This lizard, which is native from Africa, is one of the five invasive reptile species presently known to occur in Brazil (Rocha et al. 2011) and has continuously invaded natural environments in Brazil during the past 70 years (Rocha et al. in press).

Our data highlight the singularity of the LMNP in the context of the biomes that surround it, and also demonstrate the importance of actions to improve conservation of reptiles that live in both sand dunes and restingas in LMNP. Currently, restingas comprise only about 20% of the total area protected by the park, but most reptile species live in restinga mesohabitats. Thus, we suggest the addition of restinga areas adjacent to the park (buffer zones) to be incorporated into the national park, which is a fully protected conservation unit in the Brazilian system of conservation units. This would be the most effective way to protect the biodiversity of reptiles in the restinga areas in that region. Moreover, regarding the sand dunes areas; we suggest an improvement in the security at the LMNP to prevent the illegal use of off-road vehicles inside the park territory, the promotion of actions to monitor the activities of sea turtles at the coast of LMNP, and the implementation of an effective strategy to protect the Brazilian slider turtle. This strategy could be in the form of awareness campaigns, or even the promotion of training courses (*e. g*. tourist guides, waiters, cooks, or hotel maids) for those living in the park region. This would place local people into the tourism business, which would not only improve their economical capacity, but also reduce their need to use *Trachemys adiutrix* as a food item.

**Figure 5.**
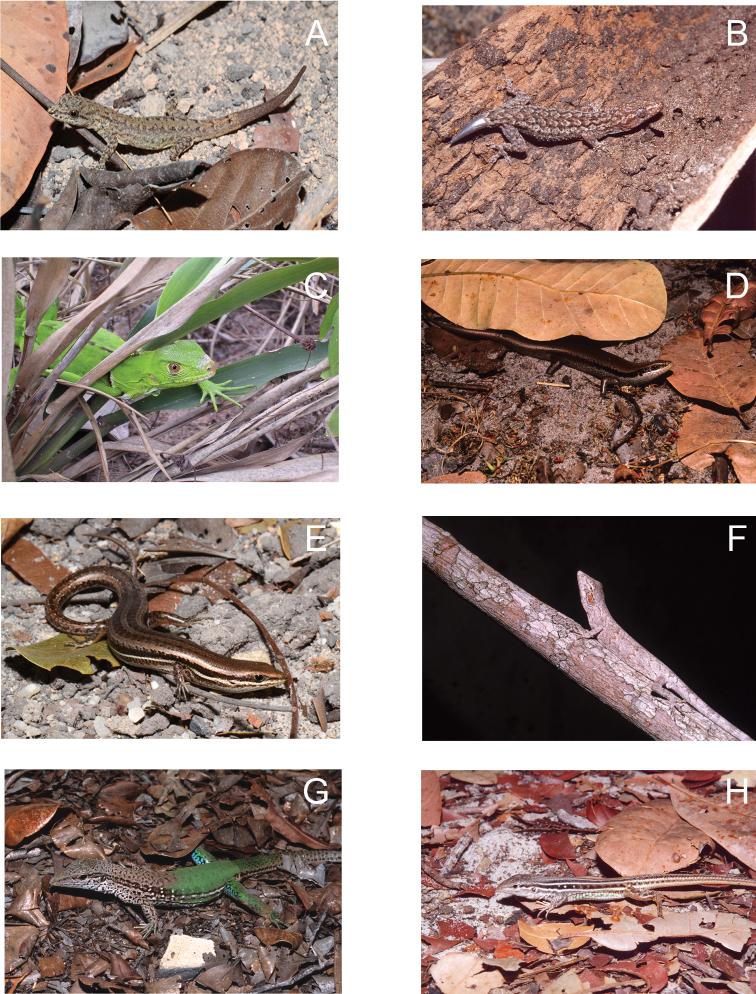
**A** *Gonatodes humeralis* (female) **B** *Hemidactylus mabouia* **C** *Iguana iguana* (juvenile) **D** *Varzea bistriata* **E** *Brasiliscincus heathi* **F** *Polychrus acutirostris* **G** *Ameiva ameiva* **H** *Ameivula ocellifera* from Lençóis Maranhenses National Park, Maranhão State, Northeastern Brazil. Photos by J. P. Miranda.

**Figure 6.**
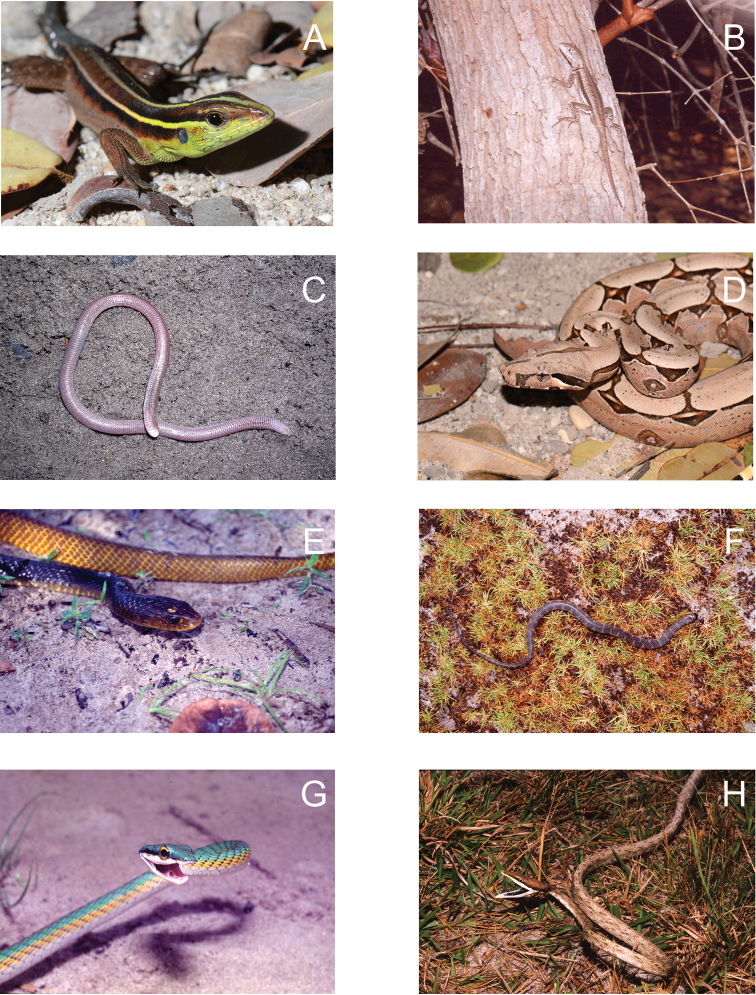
**A** *Kentropyx calcarata* **B** *Tropidurus hispidus* **C** *Amphisbaena ibijara* **D** *Boa constrictor* **E** *Drymarchon corais* **F** *Helicops angulatus* **G** *Leptophis ahaetulla* **H** *Oxybelis aeneus* from Lençóis Maranhenses National Park, Maranhão State, Northeastern Brazil. Photos by J. P. Miranda.

**Figure 7.**
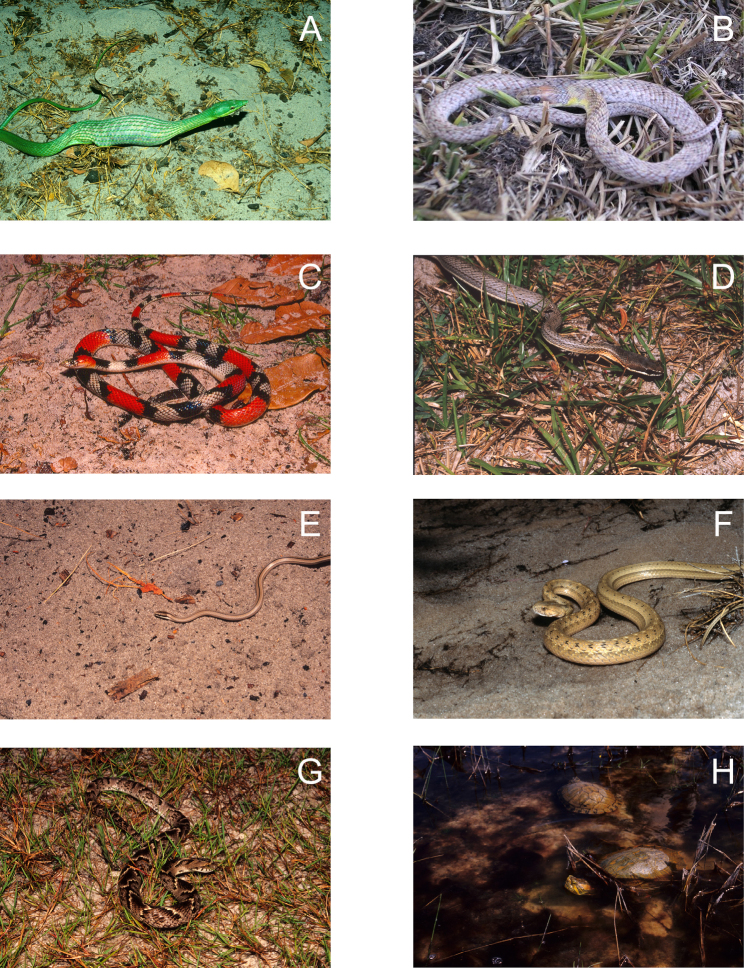
**A** *Oxybelis fulgidus* **B** *Erythrolamprus poecilogyrus* **C** *Oxyrhopus trigeminus* **D** *Philodryas nattereri* **E** *Psomophis joberti* **F** *Thamnodynastes hypoconia* **G** *Xenodon merremii* **H** *Trachemys adiutrix* (two individuals in a freshwater lagoon) from Lençóis Maranhenses National Park, Maranhão State, Northeastern Brazil. Photos by J. P. Miranda.

## Appendix

### Specimens examined

Voucher specimens of the present study were deposited in the Coleção Herpetológica “Claude d’Abbeville” (CHMA) at Universidade Federal do Maranhão, Chapadinha, Maranhão, Brazil.

**OrderSAURIA**

**Family Sphaerodactylidae**

*Gonatodes humeralis* – CHMA 554.

**Family Gekkonidae**

*Hemidactylus mabouia* – CHMA 538, 542, 545, 555, 557, 561.

**Family Mabuyidae**

*Varzea bistriata* – CHMA 503.

*Brasiliscincus heathi* – CHMA 504, 505, 549.

**Family Polychrotidae**

*Polychrus acutirostris* – CHMA 551, 556.

**Family Teiidae**

*Ameiva ameiva* – CHMA 507, 509, 511, 535, 548, 569.

*Ameivula ocellifera* – CHMA 506, 508, 510, 515, 517, 520-524, 571, 574.

*Kentropyx calcarata* – CHMA 512.

*Tupinambis teguixin* – CHMA 567.

**Family Tropiduridae**

*Tropidurus hispidus* – CHMA 558-560, 562-564.

**OrderAMPHISBAENIA**

**Family Amphisbaenidae**

*Amphisbaena ibijara* – CHMA 516, 518.

*Amphisbaena vermicularis* – CHMA 513, 519.

**OrderSERPENTES**

**Family Colubridae**

*Chironius flavolineatus* – CHMA 537, 572.

*Drymarchon corais* – CHMA 576.

*Mastigodryas bifossatus* – CHMA 566.

*Oxybelis aeneus* – CHMA 541.

*Spilotes pullatus* – CHMA 570.

*Tantilla melanocephala* – CHMA 575.

**Family Dipsadidae**

*Helicops angulatus* – CHMA 529, 530, 573.

*Hydrodinastes gigas* – CHMA 568.

*Erythrolamprus poecilogyrus* – CHMA 536, 539, 543, 552.

*Leptodeira annulata* – CHMA 528

*Oxyrhopus trigeminus* – CHMA 525-527.

*Philodryas nattereri* – CHMA 534, 544.

*Philodryas olfersii* – CHMA 540.

*Psomophis joberti* – CHMA 500, 501, 553.

*Taeniophallus occipitalis* – CHMA 550.

*Thamnodynastes hypoconia* – CHMA 531-533.

*Xenodon merremii* – CHMA 546.

**Family Elapidae**

*Micrurus ibiboboca* – CHMA 547.

**OrderTESTUDINES**

**Family Emydidae**

*Trachemys adiutrix* – CHMA 565.
